# A rare case of bilateral renal angiomyolipoma: Radiological findings of tuberous sclerosis complex

**DOI:** 10.1002/ccr3.7368

**Published:** 2023-05-20

**Authors:** Sajjad Ali, Ali Salim Lalani, Deblina Mukherjee, Kayhan Nasir Hashmi

**Affiliations:** ^1^ Department of Internal Medicine Ziauddin Medical University Karachi Pakistan; ^2^ Department of Medicine St. George's University School of Medicine West Indies Grenada

**Keywords:** radiology, renal angiomyolipoma, tuberous sclerosis complex

## Abstract

**Key Clinical Message:**

Renal angiomyolipoma is a type of benign hamartoma that may occur sporadically or be associated with tuberous sclerosis complex. Due to their distinct appearance, CT, MRI, or sonography are typically used to diagnose AMLs.

**Abstract:**

The uncommon benign hamartoma known as renal angiomyolipoma (AML) linked with tuberous sclerosis has a poor prognosis and potentially fatal side effects. Due of their distinct appearance, computed tomography (CT), magnetic resonance imaging (MRI), or sonography are typically used to diagnose AMLs.

## 1 CASE DESCRIPTION

A 20‐year‐old female patient with known comorbid epilepsy and mild intellectual disability complained of recent hematuria and one‐year‐long abdominal discomfort. The discomfort began gradually, was limited to the left lumbar area, and was made worse by physical activity and reduced fluid intake. Moreover, she noticed dark urine 2 days back, visible throughout the flow. Intermittent burning sensation during micturition and sporadic pain over the hypogastric region were present.

**FIGURE 1:**
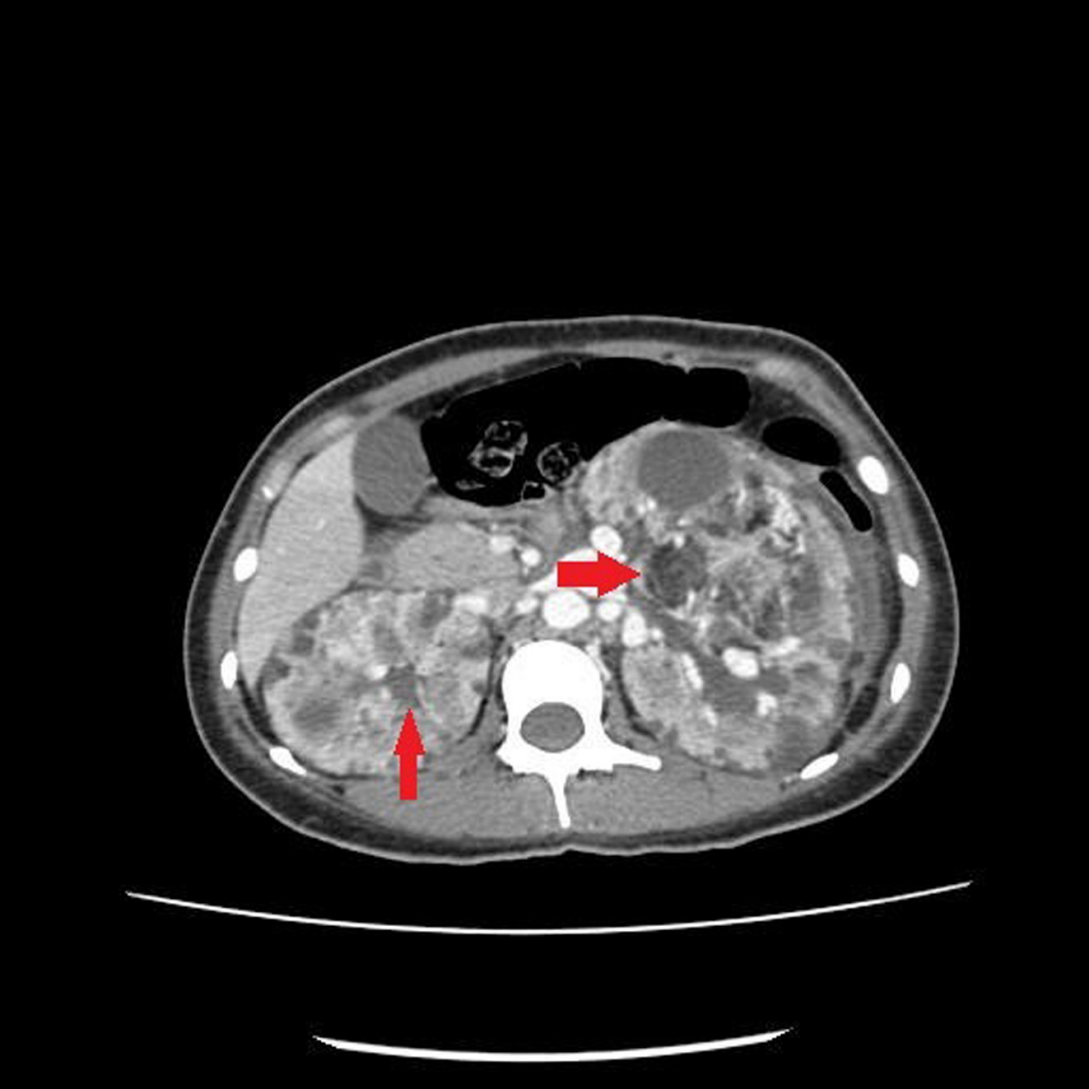
There are multiple varying sizes mixed density lesions in both kidneys, the largest one in the left kidney measures approximately 8.3 × 7.6 × 14.3 cm in APxTRxCC dimensions (Red Arrow).

**FIGURE 2:**
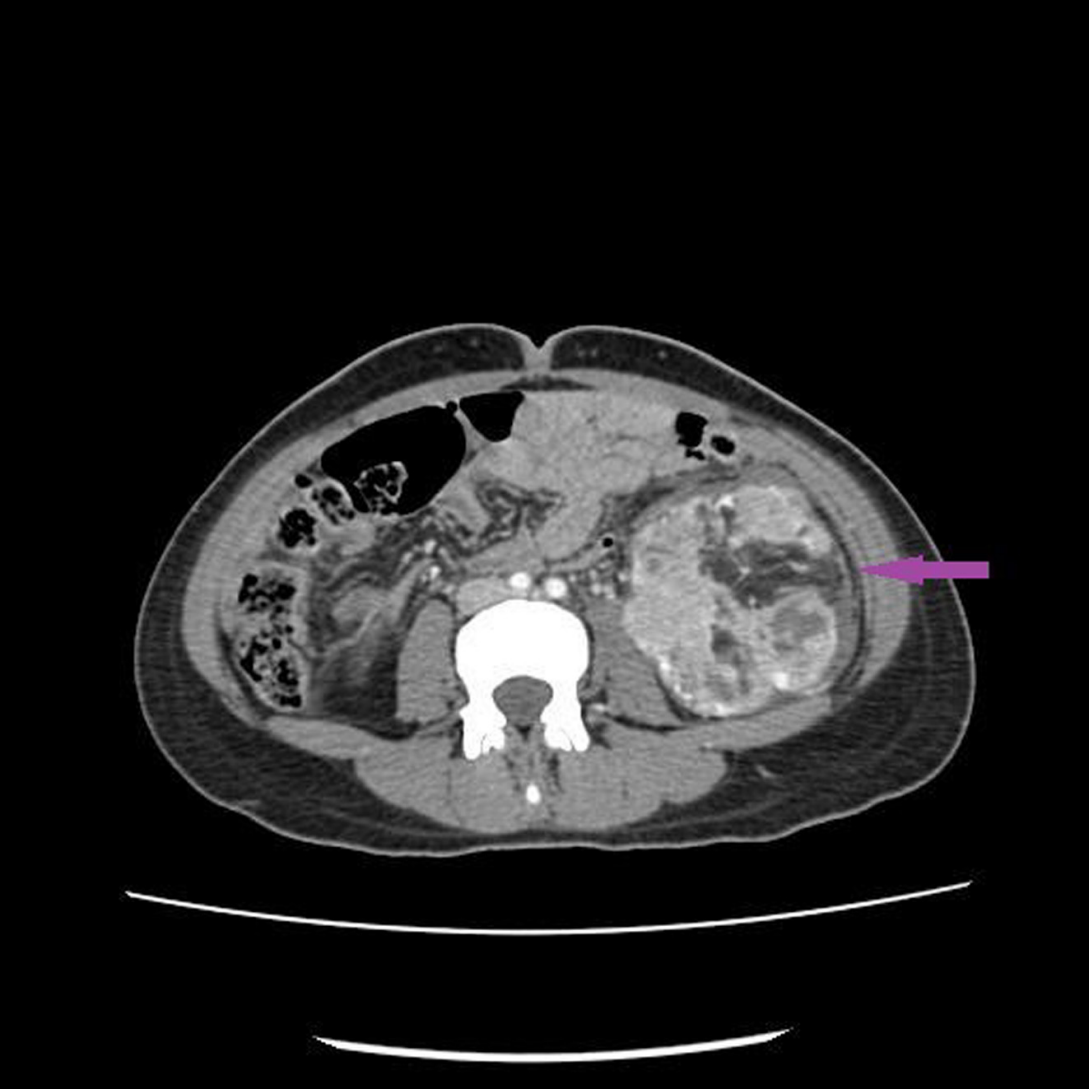
The lesion shows occasional focal breaches in its capsule resulting in the formation of blood clots within the pelvicalyceal system of the left kidney (Purple Arrow).

A contrast‐enhanced computed tomography (CE‐CT) was done (Figure 1). Both kidneys were enlarged. There were multiple varying sizes of mixed‐density lesions in both kidneys, showing areas of fat attenuation, soft tissue component, and enlarged vessels, the largest one in the left kidney measured approximately 8.3 × 7.6 × 14.3 cm in APxTRxCC dimensions. The described imaging features suggested bilateral renal AML with possible rupture in the largest left renal AML (Figure 2).

Additionally, magnetic resonance imaging (MRI) of the brain was performed, revealing subependymal calcification, and cortical tubers.

The definitive diagnosis of tuberous sclerosis complex was reached after reviewing her medical history and radiological findings.

## 2 DISCUSSION

A benign hamartoma known as renal angiomyolipoma (AML) may develop sporadically or be linked to tuberous sclerosis complex (TSC).^1^, ^2^, ^3^ According to reports, there are four times as many random angiomyolipoma instances as TSC cases.^3^, ^4^ Adipose tissue, blood arteries, and smooth muscle make up the “triphasic” structure of AML. Signs and symptoms vary by size; small renal AMLs are often asymptomatic, while large renal AMLs have a mass effect on healthy kidney tissue, which, if not treated, can lead to chronic kidney disease (CKD). Using abdominal CT or MRI, one can quickly diagnose AML due to the tumor's histologic fat composition. However, a CT scan is requested in a patient with tuberous sclerosis to search for probable renal AMLs. Sporadic AMLs are typically discovered by coincidence.

Despite the abundance of renal AML, aneurysm formation, or rupture seldom occurs in those less than 4 cm in size. Imaging enables the close monitoring of small lesions. AMLs larger than 4 cm have a higher propensity for acute bleeding; as a result, even while asymptomatic, they can benefit from angiography and embolization as opposed to conventional surgical therapeutic alternatives. Acute arterial bleeding usually necessitates prompt medical attention when symptomatic.

In conclusion, diagnostic imaging is the key to detecting renal AML. AMLs are typically not diagnosed with a biopsy; however, in challenging or complex instances, such as fat‐invisible renal mass lesions, a biopsy may be required to rule out malignancy.

## AUTHOR CONTRIBUTIONS

**Sajjad Ali:** Conceptualization; data curation; formal analysis; project administration; resources; supervision; visualization; writing – original draft; writing – review and editing. **Ali Salim Lalani** Formal analysis; investigation; methodology; software; writing – original draft. **Deblina Mukherjee:** Data curation; funding acquisition; validation; visualization; writing – review and editing. **Kayhan Nasir Hashmi:** Data curation; methodology; resources; validation; visualization.

## FUNDING INFORMATION

None.

## CONFLICT OF INTEREST STATEMENT

The authors declare that they have no competing interests.

## ETHICS STATEMENT

The study was conducted in accordance with the Declaration of Helsinki. The paper is exempt from ethics committee approval as only one case was reported.

## CONSENT

The patient gave written consent for their personal or clinical details and any identifying images to be published in this study.

## Data Availability

All data generated or analyzed during this study are included in this published article [and its supplementary information files].
